# Postpartum Psychosis as a Precursor to Schizophrenia: A Comprehensive Review

**DOI:** 10.7759/cureus.68451

**Published:** 2024-09-02

**Authors:** Rishitha Kotla, Pradeep S Patil, Isha Ahluwalia

**Affiliations:** 1 Psychiatry, Jawaharlal Nehru Medical College, Datta Meghe Institute of Higher Education and Research, Wardha, IND

**Keywords:** clinical management, mental health, early detection, risk factors, schizophrenia, postpartum psychosis

## Abstract

Postpartum psychosis (PP) is a rare and severe mental health disorder occurring shortly after childbirth, characterized by symptoms such as delusions, hallucinations, and intense mood swings. This review examines the potential link between PP and the later development of schizophrenia, a chronic psychiatric condition that typically emerges in late adolescence or early adulthood. By reviewing existing literature and analyzing epidemiological and clinical data, this review aims to clarify whether PP can be a precursor to schizophrenia. Findings suggest that while the transition from PP to schizophrenia is not inevitable, there is an increased risk, with some studies indicating that a subset of women with PP may develop a chronic psychotic disorder later on. This underscores the importance of early detection, ongoing monitoring, and targeted interventions. The review emphasizes the need for improved diagnostic practices and preventive measures to better manage PP and its potential long-term effects. Enhanced understanding of this relationship can inform more effective treatment strategies and support better mental health outcomes for new mothers. Future research should focus on refining risk assessment tools, exploring underlying mechanisms, and developing comprehensive management approaches to address the challenges associated with PP and its potential progression to schizophrenia.

## Introduction and background

Postpartum psychosis (PP) is a severe mental health condition that emerges shortly after childbirth, characterized by a sudden onset of psychotic symptoms such as delusions, hallucinations, severe mood swings, and disorganized thinking [1]. Though it is a rare condition, affecting approximately 1 to 2 in 1,000 new mothers, its impact is profound and immediate, often necessitating urgent intervention to safeguard both the mother and her infant [2]. This disorder contrasts with schizophrenia, a chronic mental illness typically manifesting in late adolescence or early adulthood. Schizophrenia is marked by persistent symptoms, including hallucinations, delusions, and impaired cognitive function, affecting about 1% of the global population. Despite the distinct onset and progression of these two conditions, they share some common features, particularly in the realm of psychosis, which underscores the need to understand their potential interconnection [3].

The prevalence of PP is relatively low compared to other postpartum mood disorders, such as postpartum depression. However, its potential link to chronic psychiatric conditions like schizophrenia is a significant concern. Research suggests that women who experience PP are at an increased risk of developing schizophrenia or other severe psychiatric disorders later in life [4]. Although not all women with PP will progress to schizophrenia, studies indicate that up to 25% may face a transition to a more chronic psychotic illness. This elevated risk highlights the importance of monitoring and understanding the progression from PP to more enduring conditions [5].

The primary aim of this review is to investigate the relationship between PP and the subsequent development of schizophrenia. By synthesizing current evidence, this review seeks to determine whether PP can be considered a precursor to schizophrenia and to identify factors that may influence this progression. Understanding these dynamics is crucial for improving early detection and intervention strategies, which can significantly enhance outcomes for affected women. Additionally, this review aims to provide valuable insights for clinical practice, offering a deeper understanding of the management of PP and its long-term implications. This knowledge is essential for developing more effective prevention and treatment approaches, ultimately supporting better mental health care for new mothers.

## Review

Pathophysiology and risk factors

Etiology of PP

PP is a severe psychiatric disorder that typically emerges within the first few weeks following childbirth. Its development is influenced by a combination of genetic, hormonal, and environmental factors [1]. Genetic predisposition plays a significant role in the onset of PP. Women with a personal or family history of mood disorders, particularly bipolar disorder, are at an elevated risk. Research indicates that approximately 40-50% of women experiencing PP have a history of bipolar disorder or related psychotic disorders. Furthermore, studies show that 20-30% of women with known bipolar disorder will encounter PP after childbirth, underscoring the importance of genetic factors in this condition [6]. Hormonal changes also play a crucial role in the onset of PP. The postpartum period is marked by rapid fluctuations in hormone levels, especially estrogen and progesterone. These hormonal shifts can significantly affect mood and behavior, potentially triggering psychotic episodes in individuals who are already predisposed to mood disorders. The interaction between these hormonal changes and genetic vulnerabilities can create a perfect storm for developing PP [6]. Environmental factors, such as psychosocial stressors, further increase the risk of developing PP. Factors including lack of social support, sleep deprivation, and the overwhelming demands of new motherhood can contribute to heightened stress levels. Additionally, obstetric complications during pregnancy or childbirth, such as preeclampsia, can elevate the likelihood of developing PP. These complications may lead to increased stress and hormonal imbalances, which can provoke psychotic symptoms in susceptible individuals [7].

Clinical Features of PP

PP is marked by a range of severe symptoms that can profoundly impair a woman's ability to function and pose significant risks to both her and her infant's safety. Common symptoms include delusions, hallucinations, mood swings, cognitive impairment, and altered states of consciousness. Delusions may present as paranoid, grandiose, or bizarre beliefs, representing a substantial departure from the individual's prior thoughts and behaviors. Women may also experience auditory or visual hallucinations, which can be highly distressing and disorienting [4]. Mood swings are a key feature of PP, with individuals experiencing dramatic fluctuations, including episodes of mania or severe depression. Cognitive impairment, which involves confused thinking and disorganized behavior, can severely impact daily functioning. Additionally, some women may experience altered states of consciousness, affecting their perception of reality and complicating their condition further [8]. Diagnosis of PP is generally made through clinical assessment guided by specific diagnostic criteria. Symptoms typically emerge within the first one to four weeks after childbirth. The severity of the symptoms, including severe mood disturbances and psychotic features, helps differentiate PP from other postpartum mood disorders, such as postpartum depression. Additionally, the symptoms must significantly impair the woman’s ability to perform daily activities and pose risks to her safety and that of her child [9]. Given the potential for severe outcomes, including suicide and infanticide, early recognition and intervention are critical for women experiencing PP. Treatment generally involves a combination of pharmacological and non-pharmacological approaches to ensure the safety and well-being of both mother and child [10].

Schizophrenia

Etiology

Schizophrenia is a complex mental disorder with a multifactorial etiology that includes genetic, neurodevelopmental, and environmental factors. Genetic predisposition is a significant contributor, with heritability estimates around 80%. This suggests that individuals with a family history of schizophrenia are at a higher risk of developing the disorder. Schizophrenia’s genetic architecture is polygenic, involving both common variants with minor effects and rare, damaging variants. Research indicates that the concordance rate for schizophrenia in monozygotic twins is approximately 45%, highlighting the substantial role of genetics in the disorder’s development [11]. In addition to genetic factors, neurodevelopmental influences are crucial for understanding schizophrenia. The neurodevelopmental hypothesis posits that disruptions during critical periods of brain development can contribute to the onset of the disorder. These disturbances may be influenced by genetic predispositions and environmental stressors, leading to alterations in brain connectivity and function. Environmental factors such as prenatal exposure to infections, obstetric complications, childhood trauma, and psychosocial stressors have been associated with an increased risk of developing schizophrenia. These environmental influences can interact with genetic vulnerabilities, further heightening the risk of onset [12].

Clinical Features

**Figure 1:**
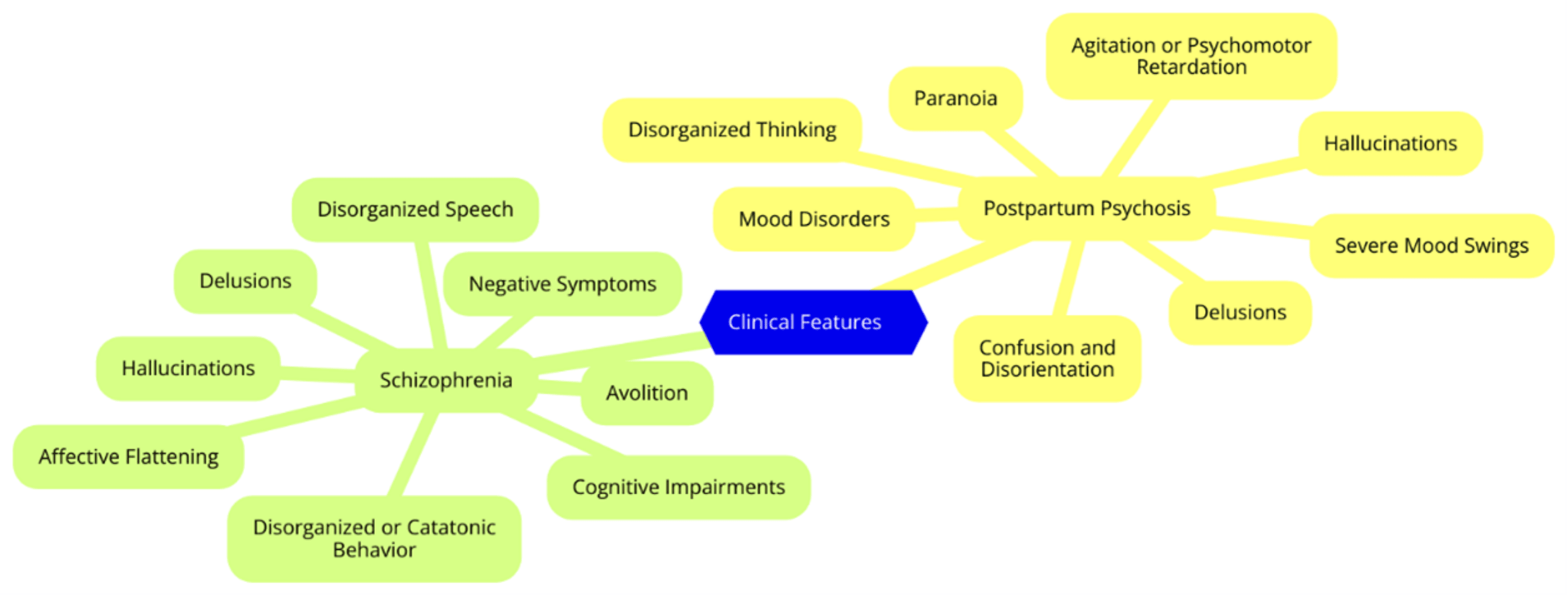
Clinical Features of Postpartum Psychosis and Schizophrenia Image Credit: Dr. Kotla Rishitha

Schizophrenia is characterized by a wide range of symptoms that are typically categorized into positive, negative, and cognitive domains. Positive symptoms include hallucinations, delusions, and disorganized thinking or speech, reflecting an excess or distortion of normal cognitive functions. Negative symptoms involve deficits in emotional responses and behaviors, such as flat affect, anhedonia, and social withdrawal. Cognitive symptoms include attention, memory, and executive function impairments, significantly affecting daily functioning and overall quality of life [12]. The diagnosis of schizophrenia is based on criteria outlined in the Diagnostic and Statistical Manual of Mental Disorders, Fifth Edition (DSM-5), which requires the presence of at least two of the following symptoms for a significant portion of one month: delusions, hallucinations, disorganized speech, grossly disorganized or catatonic behavior, and negative symptoms. These symptoms must also cause significant impairment in social or occupational functioning. The complexity of these symptoms requires a thorough approach to diagnosis and treatment to ensure that individuals receive appropriate care and support [13]. Clinical features of PP and schizophrenia are illustrated in Figure 1.

Overlap and Transition: PP to Schizophrenia

The potential mechanisms linking PP to schizophrenia are of significant interest due to their shared symptomatology and underlying vulnerabilities. One key aspect is genetic predisposition; women with a history of bipolar disorder or schizophrenia are at an increased risk of developing PP. This genetic vulnerability may also contribute to the later onset of schizophrenia, suggesting a continuum of risk across various psychiatric disorders [14]. Neurodevelopmental pathways further elucidate the relationship between PP and schizophrenia. Hormonal changes during and after pregnancy may exacerbate pre-existing neurodevelopmental vulnerabilities, leading to the onset of psychotic symptoms in predisposed individuals. Significant stressors, including hormonal fluctuations, altered sleep patterns, and the demands of new motherhood, mark the postpartum period. These factors can act as environmental triggers that precipitate psychosis in those with genetic predispositions, potentially leading to a transition from PP to a more chronic psychotic disorder such as schizophrenia [15]. Additionally, the overlapping symptoms of PP and schizophrenia, such as hallucinations and delusions, can complicate both diagnosis and treatment. Recognizing the continuum between these disorders is crucial for early intervention and effective management strategies. By understanding the interplay of genetic, neurodevelopmental, and environmental factors, clinicians can better identify at-risk individuals and provide timely support, ultimately improving outcomes for those affected by these severe psychiatric conditions [16].

Epidemiological evidence

Incidence of Schizophrenia Following PP

The relationship between PP and the subsequent development of schizophrenia is an area of ongoing investigation. Although PP is primarily associated with bipolar disorder, direct evidence linking it to schizophrenia remains limited. Longitudinal studies provide important insights into this connection. For example, a Swedish population-based study found that among a large cohort of women, only a small fraction - merely 6 out of 610,047 - who had no prior psychiatric hospitalization were later hospitalized for schizophrenia during the postpartum period after their first birth. This finding suggests that the risk of developing schizophrenia following PP is not significantly elevated [16]. Furthermore, another significant study indicated that approximately 50% of women who experience PP have no prior psychiatric history, suggesting that many cases occur in women who may not progress to chronic psychotic disorders such as schizophrenia. Additionally, it has been noted that women with schizophrenia might experience the onset of psychotic symptoms later in the postpartum period compared to those with bipolar disorder. However, the distribution of postpartum psychotic episodes by week appeared similar across various diagnostic categories, suggesting that the timing of onset may not differ substantially among these groups [14]. In terms of risk assessment, estimates suggest that a significant majority - 72% and 88% - of PP cases occur in women with bipolar disorder or schizoaffective disorder, with only about 12% associated with schizophrenia. One study found that the incidence of PP was notably higher (9.24%) in women with a history of psychiatric hospitalization before delivery, compared to just 0.04% in those without such histories. This further underscores that the majority of PP cases are more likely linked to mood disorders rather than schizophrenia [17].

Comparative Analysis: Differences and Similarities in Incidence and Progression of PP

PP is a rare but severe mental health condition that can develop shortly after childbirth. Understanding its incidence and progression is essential for effective management and prevention [18]. The reported incidence of PP ranges from 0.89 to 2.6 per 1,000 births across various studies. Considering broader definitions and extended follow-up periods, some research reports prevalence rates of up to 5 per 1,000 births [19]. A systematic review indicates that the most consistent estimates fall within the 1 to 2 per 1,000 births range, aligning with findings from multiple cohort studies. Variations in incidence rates are attributed to differences in study methodologies, definitions of PP, and assessment timeframes [20]. Some studies focus on psychosis within the initial weeks postpartum, while others extend the observation period to several months, resulting in discrepancies in reported rates. Smaller sample sizes often show higher incidence rates, suggesting that larger studies may provide more reliable estimates. Women with a history of bipolar disorder or previous PP episodes are at significantly increased risk, with estimates suggesting that one in four births in women with bipolar disorder may be affected [1]. Typically, PP onset occurs within 10 to 19 days after childbirth, with approximately 90% of cases presenting within the first four weeks postpartum. This timing contrasts with other psychotic disorders, where onset may not be as closely linked to a specific triggering event like childbirth [21]. Most women with PP do not have a prior history of mental illness; however, many experience relapses of mood disorders or psychosis later in life, often unrelated to subsequent pregnancies. This suggests that PP may act as a precursor to more chronic psychiatric conditions, particularly in those with underlying vulnerabilities. The severity of PP includes a high risk of suicide and infanticide, making immediate clinical intervention crucial. Fortunately, early recognition and treatment often lead to full remission [4]. Despite its relatively low incidence, PP is associated with significant risks and potential long-term psychiatric implications. The variability in reported rates underscores the need for standardized definitions and assessment methods to enhance comparability across studies [22]. Understanding the progression patterns of PP, particularly its relationship with bipolar disorder, is vital for developing effective treatment strategies and preventive measures. The literature emphasizes the critical need for early diagnosis and intervention to mitigate severe outcomes, highlighting the importance of awareness among healthcare providers and support systems for new mothers [18].

Clinical Presentation and Diagnosis

PP is a severe psychiatric condition that typically emerges with a rapid onset of psychotic symptoms within the first two to four weeks after childbirth. Healthcare professionals and caregivers must recognize early warning signs to ensure timely diagnosis and intervention. Prominent symptoms of PP include paranoid, grandiose, or bizarre delusions. Women experiencing PP may hold firmly to delusions not grounded in reality, such as believing their baby is in imminent danger or that they possess special powers [18]. Additionally, mood swings that range from mania to severe depression are common, with affected women fluctuating between elation and profound sadness. Confused thinking and disorganized behavior are also characteristic of PP, making it difficult for the individual to concentrate, make decisions, or perform daily tasks. Hallucinations can also occur, including auditory, visual, or olfactory disturbances. Furthermore, insomnia and a reduced need for sleep are frequently reported. These symptoms significantly depart from the woman's baseline functioning and necessitate immediate medical attention [23]. Although PP can resemble symptoms of schizophrenia, differentiating between the two conditions is crucial. Most women with PP do not have a prior history of schizophrenia, and their psychotic episodes often resolve with appropriate treatment. However, diagnosing PP can be challenging due to several factors [24]. The rapid onset of symptoms shortly after childbirth may complicate the distinction from other postpartum mood disorders. Additionally, symptom overlap can make identifying the underlying condition difficult. Many women with PP have an underlying bipolar disorder or schizoaffective disorder rather than schizophrenia, adding to the diagnostic complexity. The psychotic nature of PP may also impair insight into the illness, making it harder for women to recognize and report their symptoms accurately [25]. Early detection and risk assessment are vital for effective intervention in PP. Various screening and assessment tools can aid healthcare professionals in identifying at-risk women and providing appropriate care. The Edinburgh Postnatal Depression Scale (EPDS) is primarily used to screen for postpartum depression but can also indicate risk for PP, as some questions address psychotic symptoms [26]. The Structured Clinical Interview for DSM-5 (SCID-5) is a comprehensive diagnostic tool that can help distinguish PP from other psychiatric disorders. Additionally, the Postpartum Psychosis Prediction Scale evaluates risk factors such as personal or family history of bipolar disorder or previous episodes of PP. Regular monitoring by healthcare providers, including obstetricians, midwives, and mental health specialists, is crucial for the early detection and management of women at high risk for PP [27].

Management and Treatment

The management of PP necessitates a comprehensive approach that integrates pharmacological treatments with psychotherapeutic interventions. Antipsychotic medications are typically the first line of treatment for alleviating acute psychotic symptoms such as delusions and hallucinations [4]. Commonly prescribed antipsychotics include olanzapine, risperidone, and quetiapine. Mood stabilizers, such as lithium, valproate, and lamotrigine, are also essential for regulating mood swings and preventing the recurrence of episodes. If significant depressive symptoms are present, antidepressants may be added, often in combination with a mood stabilizer. Benzodiazepines may be used temporarily to manage agitation and insomnia [28]. Psychological therapies are crucial in the recovery process. Cognitive-behavioral therapy (CBT) is effective in helping patients address negative thought patterns and behaviors. Counseling and support offer emotional assistance and aid patients in processing their experiences. Engaging partners, family, and friends in the care plan is vital, as is connecting with support groups and peer support workers [28]. Preventive strategies are key to reducing the risk of PP and supporting recovery. Regular psychiatric assessments and home visits by a multidisciplinary team during the postpartum period are essential for monitoring and follow-up. For high-risk patients, continuing mood stabilizers like lithium after delivery can help prevent relapse. Strong support systems, including partners, family, friends, and community resources, play an invaluable role in recovery [29]. Although PP often presents as an acute episode of bipolar disorder, there is a risk of developing chronic psychotic disorders, such as schizophrenia, in some cases. Prompt treatment of PP is critical to prevent the progression of schizophrenia. If symptoms persist or worsen despite initial treatment, it may be necessary to adjust the treatment plan and consider a diagnosis of schizophrenia. Ongoing monitoring and tailored treatment are essential for patients who develop schizophrenia following PP to ensure effective long-term management [30]. With timely and appropriate treatment, most women with PP make a full recovery. However, the risk of recurrence in future pregnancies remains high, highlighting the need for preventive strategies and long-term management. A multidisciplinary approach involving psychiatrists, psychologists, social workers, and community support is crucial to providing comprehensive care for women with PP and preventing the potential transition to schizophrenia [31]. Management and treatment strategies for PP and considerations for transition to schizophrenia are detailed in Table 1.

**Table 1:** Management and Treatment Strategies for Postpartum Psychosis and Considerations for Transition to Schizophrenia

Category	Management and Treatment for Postpartum Psychosis	Considerations for Transition to Schizophrenia
Pharmacological Treatments	Antipsychotics: Effective in managing acute psychotic symptoms. Commonly used antipsychotics include Olanzapine, Which helps alleviate delusions and hallucinations. Quetiapine: Used for its sedative and antipsychotic properties. Risperidone: Reduces symptoms of psychosis and stabilizes mood. Mood Stabilizers: Used if mood instability is prominent. Lithium: Effective for mood stabilization, particularly if bipolar symptoms are present. Valproate: Alternative for mood stabilization. Antidepressants: These may be prescribed if depressive symptoms are significant.	Long-Term Antipsychotics: For ongoing management if transitioning to schizophrenia. Clozapine: Considered for treatment-resistant schizophrenia. Aripiprazole: Used for its efficacy in managing chronic psychotic symptoms. Adjusting Doses: Treatment regimens may need adjustment based on efficacy and side effects. Monitoring: Regular psychiatric assessments to monitor treatment response and manage side effects.
Psychotherapeutic Interventions	Cognitive Behavioral Therapy (CBT): Helps address cognitive distortions and provides coping strategies for managing psychotic symptoms. Family Therapy: Involves family members in treatment to improve understanding and support. Psychoeducation: Educates the mother and family about postpartum psychosis and its management.	Continued CBT: Adapted for chronic psychosis, focusing on long-term management strategies. Supportive Therapy: Ongoing therapy to help the individual manage the symptoms of schizophrenia. Psychoeducation: Continued education for the individual and family regarding schizophrenia and its management.
Monitoring and Follow-Up	Regular Psychiatric Assessments: Frequent evaluations to track progress, adjust treatment, and ensure safety. Safety Planning: Develop plans to protect the mother and infant, including crisis intervention strategies. Infant Care Support: Support for managing infant care while addressing the mother’s mental health needs.	Ongoing Psychiatric Care: Regular follow-ups to assess for signs of schizophrenia and adjust treatment as necessary. Risk Assessment: Continuous evaluation of the risk of relapse or progression to schizophrenia. Long-Term Monitoring: Implement a structured monitoring plan to address any emerging symptoms of schizophrenia.
Support Systems	Social Support: Involves family, friends, and community resources to provide emotional and practical support. Community Resources: Access to support groups, maternal mental health services, and social services. Patient Advocacy: Ensuring that the mother receives the necessary support and resources.	Integrated Care: Collaboration between mental health professionals, primary care providers, and social services for comprehensive care. Long-Term Support: Engagement with community resources and support networks tailored to the needs of individuals with schizophrenia. Patient Advocacy: Continued advocacy to ensure access to necessary services and support.
Preventive Strategies	Education and Awareness: Providing information about the symptoms of postpartum psychosis and the importance of early intervention. Pre-delivery Planning: Identifying and preparing for risk factors associated with postpartum psychosis before childbirth. Screening: Early screening for women at high risk for postpartum psychosis.	Early Intervention Programs: Implementing strategies to detect early signs of schizophrenia and initiate treatment promptly. Prevention Programs: Tailoring prevention programs for women with a history of postpartum psychosis to mitigate the risk of developing schizophrenia. Ongoing Risk Assessment: Regular evaluation of individuals with a history of postpartum psychosis to identify any signs of transitioning to schizophrenia early.

Research gaps and future directions

Current Limitations

Research on PP faces several significant gaps that hinder our understanding and treatment of this complex condition. One major limitation is the lack of standardized diagnostic criteria. PP is not officially recognized in major diagnostic manuals, such as the DSM-5 or the 10th revision of the International Classification of Diseases (ICD-10), which leads to inconsistent definitions and assessments across studies. This lack of standardization complicates comparing data and understanding the disorder's true prevalence, symptomatology, and nature. Without a unified framework, researchers struggle to draw meaningful conclusions about the condition and its treatment [31]. Additionally, research on PP is notably underrepresented in low- and middle-income countries (LMICs). Most existing studies are conducted in high-income settings, which may not accurately reflect the incidence or presentation of PP in diverse cultural contexts. This gap limits our understanding of how socioeconomic factors, cultural beliefs, and healthcare accessibility impact the experience and treatment of PP in different regions. Furthermore, many studies are characterized by small sample sizes and reliance on naturalistic designs rather than randomized controlled trials, affecting findings' generalizability and treatment recommendations' robustness [32]. Another critical limitation is the minimal involvement of stakeholders, particularly women with lived experience of PP, in the research process. Their insights and experiences are often overlooked in traditional clinical research, leading to a disconnect between research findings and the realities faced by those affected by PP. Engaging these women in research could provide valuable perspectives that enhance understanding and improve treatment approaches [32].

Emerging Research

Despite existing limitations, recent research and advancements are beginning to address some of the gaps in our understanding of PP. Emerging longitudinal studies are exploring the long-term outcomes for women who experience PP. These studies indicate that while many women recover fully, a significant proportion may later develop severe mood disorders, particularly those with underlying bipolar disorder. This underscores the importance of ongoing monitoring and support for women who have experienced PP, as they may face an increased risk of future psychiatric issues [33]. Research is also increasingly recognizing the significance of cultural perspectives in understanding PP. Preliminary findings suggest that while the core symptoms of PP may be consistent across cultures, conceptualizations and treatment approaches can vary widely. This highlights the need for culturally sensitive assessment tools and interventions tailored to the specific needs of diverse populations. By acknowledging these cultural differences, researchers can develop more effective prevention and treatment strategies [34]. Technological advancements are further contributing to the evolving research landscape. The rise of telemedicine and mobile health applications presents new opportunities for enhancing access to care and monitoring for women at risk of PP, especially in underserved areas. These technologies can facilitate timely interventions and provide continuous support, which is crucial for women experiencing mental health challenges after childbirth [35].

Recommendations for Future Research and Clinical Practice Improvements

To advance our understanding and treatment of PP, several areas for future research and clinical practice improvements are recommended. First, there is a critical need to develop standardized diagnostic tools for PP. Establishing validated, culturally sensitive assessment instruments that can be used internationally would improve diagnosis and treatment across diverse healthcare settings. A unified diagnostic framework would also facilitate more effective comparisons of research findings and contribute to advancing the field [4]. Additionally, increasing research efforts in LMICs is essential. Understanding PP's unique challenges and incidence rates in these regions could help develop tailored interventions that address specific cultural and socioeconomic contexts. Expanding research to include diverse populations will provide a more comprehensive understanding of PP and its implications [36]. Engaging stakeholders, particularly women with lived experience of PP, is another important recommendation for future research. Their insights can help shape relevant research questions and enhance the applicability of findings to real-world scenarios. Involving these women in the research process can also foster empowerment and support, which are crucial for recovery [37]. Conducting larger, multi-centre studies with diverse populations will improve the generalizability of findings and assist in identifying risk factors and effective treatments for PP. Such studies can offer a more nuanced understanding of the condition and inform best practices for clinical management [38]. Finally, research should prioritize prevention and early intervention strategies. Identifying early predictors of PP and developing preventive measures, including education for healthcare providers and expectant mothers about the signs and risks associated with PP, could significantly enhance outcomes. Focusing on these areas will deepen our understanding of PP and ultimately benefit affected women and their families [39].

## Conclusions

In conclusion, the relationship between PP and the subsequent development of schizophrenia represents a critical area of study with significant implications for both clinical practice and patient outcomes. While PP is a rare but severe condition that poses immediate risks to new mothers, its potential role as a precursor to schizophrenia underscores the need for vigilant monitoring and early intervention. The evidence suggests that while not all women with PP will transition to schizophrenia, there is a notable increased risk that warrants attention. By understanding the pathways through which PP may evolve into chronic psychotic disorders, healthcare providers can better identify at-risk individuals and implement targeted preventive strategies. Ultimately, advancing our knowledge in this field is crucial for improving diagnostic accuracy, optimizing treatment approaches, and enhancing mental health support for new mothers, thereby contributing to better long-term outcomes and quality of life.
